# Supplementary figures and images for: Does highlighting COVID-19 disparities reduce or increase vaccine intentions? evidence from a survey experiment in a diverse sample in New York State prior to vaccine roll-out
Source: PLoS One. 2022 Dec 14;17(12):e0277043. doi: 10.1371/journal.pone.0277043 (PMC9750017; doi:10.1371/journal.pone.0277043)

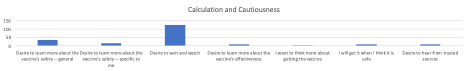

Supplement: S1 File — (ZIP) [file pone.0277043.s001.zip › S4 Fig 5.png]

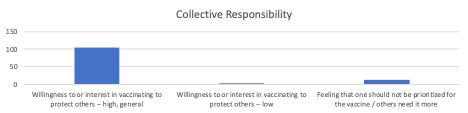

Supplement: S1 File — (ZIP) [file pone.0277043.s001.zip › S4 Fig 6.png]

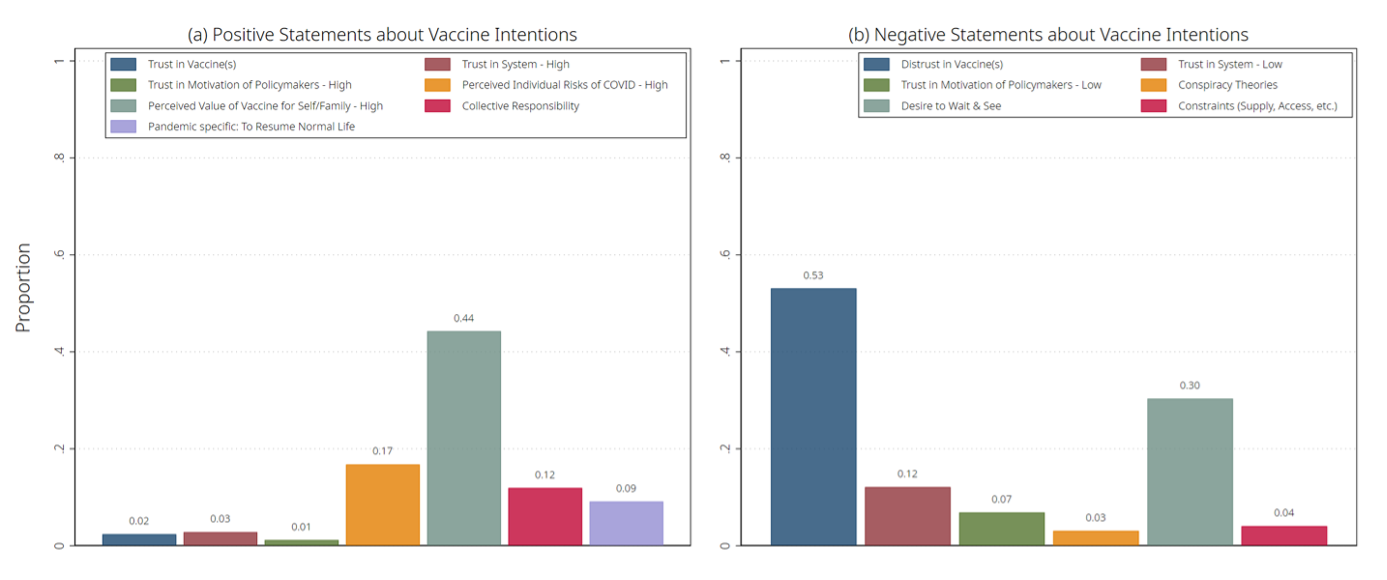

Supplement: S1 File — (ZIP) [file pone.0277043.s001.zip › S4 Fig 7.png]

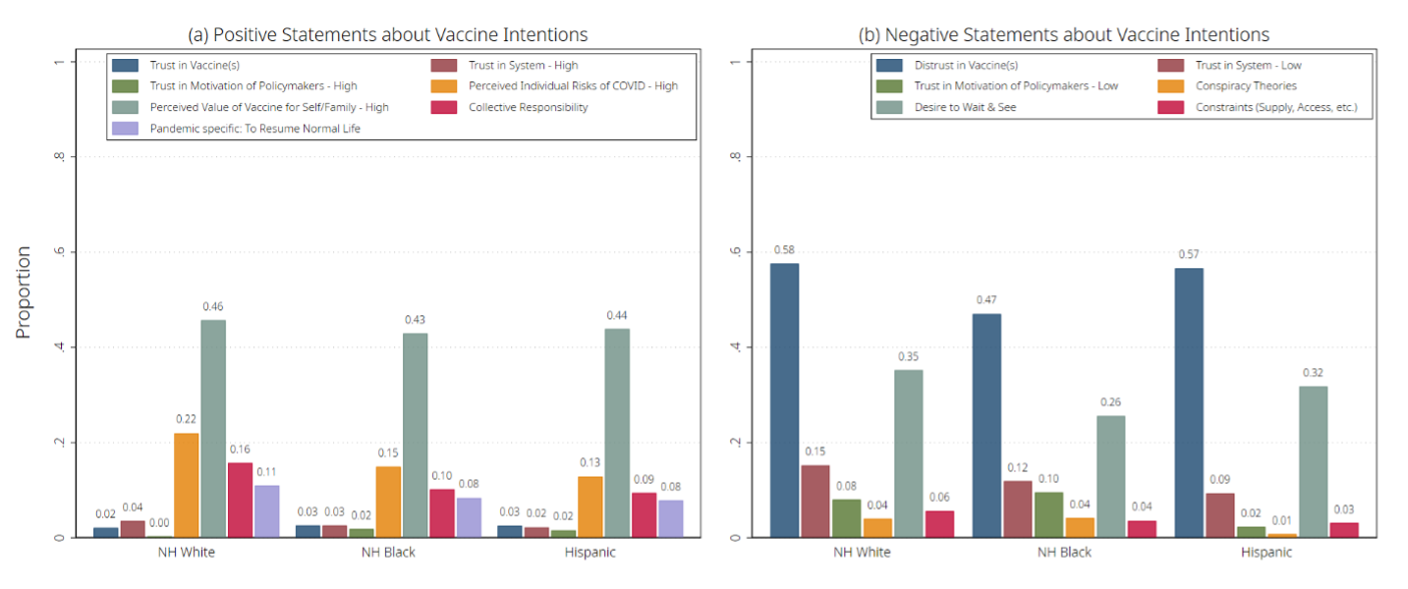

Supplement: S1 File — (ZIP) [file pone.0277043.s001.zip › S4 Fig 8.png]

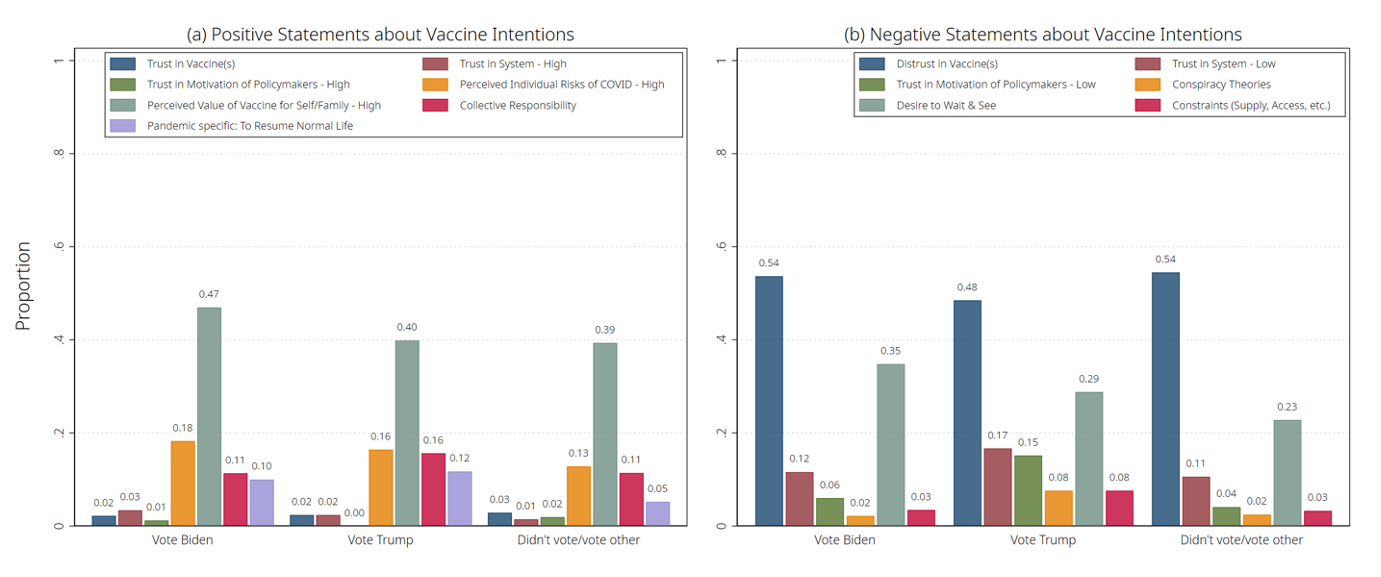

Supplement: S1 File — (ZIP) [file pone.0277043.s001.zip › S4 Fig 9.png]

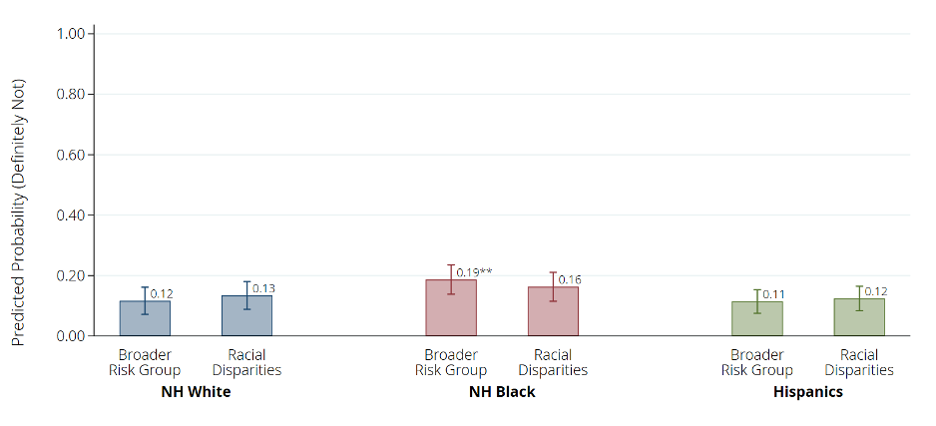

Supplement: S1 File — (ZIP) [file pone.0277043.s001.zip › S3 Fig 1 Exp_Race_Eth.png]

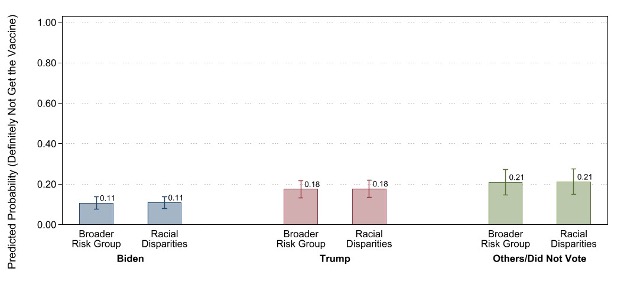

Supplement: S1 File — (ZIP) [file pone.0277043.s001.zip › S3 Fig 2 Exp_Vote_Choice.jpg]

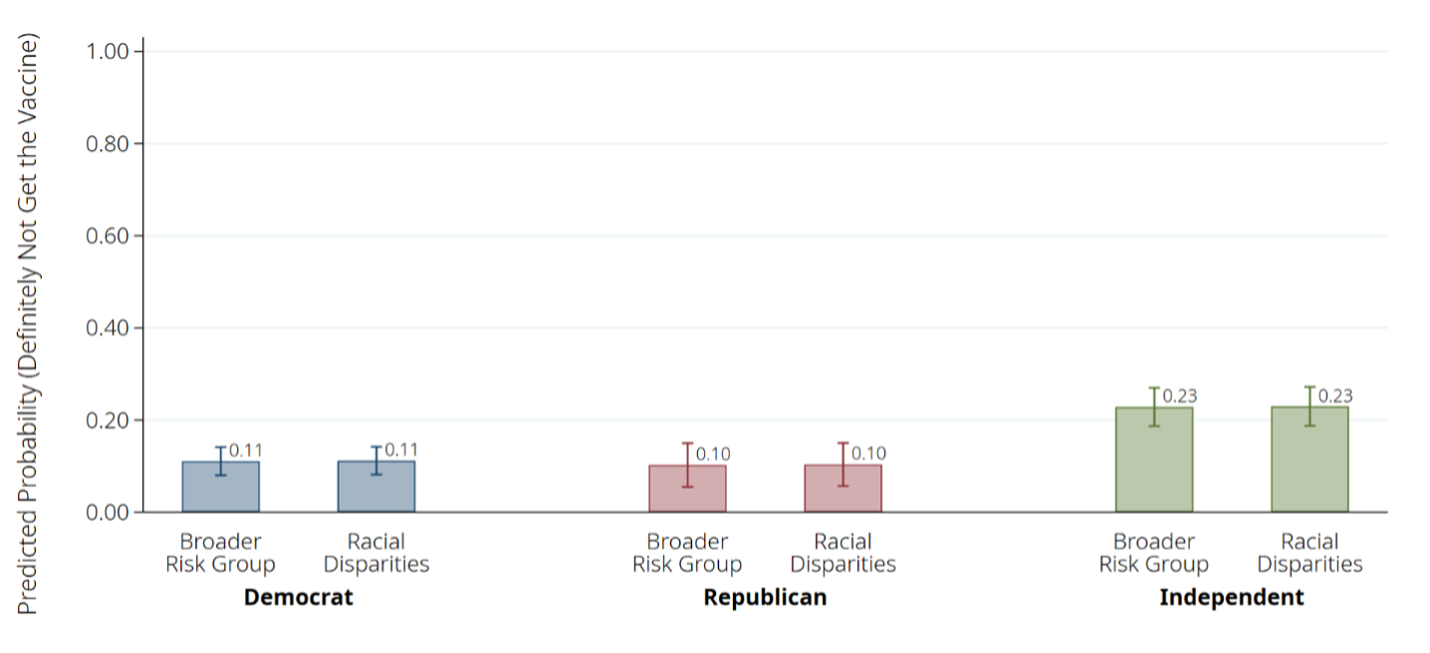

Supplement: S1 File — (ZIP) [file pone.0277043.s001.zip › S3 Fig 3 Exp_Party_ID.png]

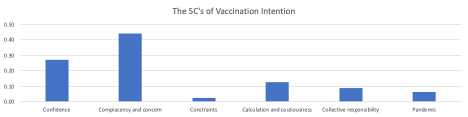

Supplement: S1 File — (ZIP) [file pone.0277043.s001.zip › S4 Fig 1.png]

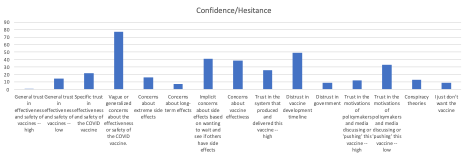

Supplement: S1 File — (ZIP) [file pone.0277043.s001.zip › S4 Fig 2.png]

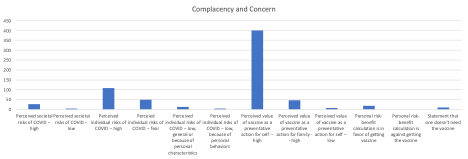

Supplement: S1 File — (ZIP) [file pone.0277043.s001.zip › S4 Fig 3.png]

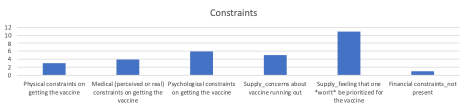

Supplement: S1 File — (ZIP) [file pone.0277043.s001.zip › S4 Fig 4.png]
