# Supplementary material for: Does highlighting COVID-19 disparities reduce or increase vaccine intentions? evidence from a survey experiment in a diverse sample in New York State prior to vaccine roll-out
Source: PLoS One. 2022 Dec 14;17(12):e0277043. doi: 10.1371/journal.pone.0277043 (PMC9750017; doi:10.1371/journal.pone.0277043)
Supplement: S2 File — (DOCX) [file pone.0277043.s002.docx]

**SUPPLEMENTAL MATERIALS**

# The full GitHub repository can be found here:

# <https://github.com/TheYongjinChoi/NYS-COVID19-Disparities-Survey>

# Supplement 1: SCALE CREATION

**S1 Table 1: Confidence in Public Institutions Scale**

Q11. How much confidence, if any, do you have that each of the following will act in the best interest of the public when it comes to researching, developing, and distributing a COVID-19 vaccine as of today?

|  | No confidence at all (1) | Very little confidence (2) | Some confidence (3) | A great deal of confidence (4) | Complete confidence (5) |
| --- | --- | --- | --- | --- | --- |
| a. Elected federal government officials under the Biden Administration |  |  |  |  |  |
| b. Federal health agencies (e.g., the FDA and CDC) |  |  |  |  |  |
| b. local public health agencies |  |  |  |  |  |
| c. Medical scientists and researchers |  |  |  |  |  |
| d. Physicians |  |  |  |  |  |
| e. U.S.-based Pharmaceutical  companies |  |  |  |  |  |
| f. Chinese-based researchers/companies |  |  |  |  |  |
| g. European-based researchers/companies |  |  |  |  |  |

//CONFIDENCE/TRUST Scale

recode VACCINE_11_1 (1=0 "no confidence") (2=1 "very little confidence") (3=2 "some confidence") (4=3 "great deal of confidence") (5=4 "complete confidence"), generate(confidence_Biden_Admin_ord)

recode VACCINE_11_2 (1=0 "no confidence") (2=1 "very little confidence") (3=2 "some confidence") (4=3 "great deal of confidence") (5=4 "complete confidence"), generate(confidence_Fed_Health_Agencies_ord)

recode VACCINE_11_3 (1=0 "no confidence") (2=1 "very little confidence") (3=2 "some confidence") (4=3 "great deal of confidence") (5=4 "complete confidence"), generate(confidence_loc_PH_Agencies_ord)

recode VACCINE_11_4 (1=0 "no confidence") (2=1 "very little confidence") (3=2 "some confidence") (4=3 "great deal of confidence") (5=4 "complete confidence"), generate(confidence_Med_Sci_Res_ord)

recode VACCINE_11_5 (1=0 "no confidence") (2=1 "very little confidence") (3=2 "some confidence") (4=3 "great deal of confidence") (5=4 "complete confidence"), generate(confidence_Physicians_ord)

recode VACCINE_11_6 (1=0 "no confidence") (2=1 "very little confidence") (3=2 "some confidence") (4=3 "great deal of confidence") (5=4 "complete confidence"), generate(confidence_Phrma_ord)

recode VACCINE_11_7 (1=0 "no confidence") (2=1 "very little confidence") (3=2 "some confidence") (4=3 "great deal of confidence") (5=4 "complete confidence"), generate(confidence_Chinese_Phrma_ord)

recode VACCINE_11_8 (1=0 "no confidence") (2=1 "very little confidence") (3=2 "some confidence") (4=3 "great deal of confidence") (5=4 "complete confidence"), generate(confidence_Euro_Phrma_ord)

generate confidence_pub_expert_scale= (confidence_Biden_Admin_ord + confidence_Fed_Hlth_Ag_ord + confidence_loc_PH_Agencies_ord + confidence_Med_Sci_Res_ord + confidence_Physicians_ord

confidence_Phrma_ord confidence_Chinese_Phrma_ord confidence_Euro_Phrma_ord

generate confidence_pubexpert_scale= (confidence_Biden_Admin_ord + confidence_Fed_Hlth_Ag_ord + confidence_loc_PH_Agencies_ord + confidence_Med_Sci_Res_ord + confidence_Physicians_ord)/5

generate confidence_pubexpert_scale2= confidence_Biden_Admin_ord + confidence_Fed_Hlth_Ag_ord + confidence_loc_PH_Agencies_ord + confidence_Med_Sci_Res_ord + confidence_Physicians_ord

recode confidence_pubexpert_scale (0=0 "no confidence") (0.1/1=1 "very little confidence") (1.1/2=2 "some confidence") (2.1/3=3 "great deal of confidence") (3.1/4=4 "complete confidence"), generate(confidence_scale)

recode confidence_scale (0 1=1), generate(confidence_scale3)

recode confidence_scale3 (1=4 "little to no confidence") (2=3 "some confidence") (3=2 "great deal confidence") (4=1 "complete confidence"), generate(lack_of_confidence)

# S1 Table 2: Cultural Cognition – 6 Item short form

### ***Individualism/Communitarianism***. People in our society often disagree about how far to let individuals go in making decisions for themselves. How strongly do you agree or disagree with the following statements:

|  | Agree Strongly (1) | Agree (2) | Neither agree nor disagree (3) | Disagree (4) | Disagree Strongly (5) |
| --- | --- | --- | --- | --- | --- |
| ***83***a. The government interferes far too much in our everyday lives |  |  |  |  |  |
| ***83***b. Sometimes government needs to make laws that protect people from hurting themselves |  |  |  |  |  |
| ***83***c. It’s not the government’s business to try to protect people from hurting themselves. |  |  |  |  |  |
| ***83***d. The government should stop telling people how to live their lives. |  |  |  |  |  |
| ***83***e. The government should do more to advance society’s goals even if that means limiting the freedom of choices of individuals. |  |  |  |  |  |
| ***83***f. Government should put limits on the choices individuals can make so they do not get in the way of what is good for society. |  |  |  |  |  |
| ***83g***. People should not do things that are disgusting, even if no one is harmed. |  |  |  |  |  |

*reverse code CULTURE_11_2 CULTURE_11_5 CULTURE_11_6

recode CULTURE_11_2 (1=5) (2=4) (3=3) (4=2) (5=1), generate(CULTURE_11_2_reverse_code)

recode CULTURE_11_5 (1=5) (2=4) (3=3) (4=2) (5=1), generate(CULTURE_11_5_reverse_code)

recode CULTURE_11_6 (1=5) (2=4) (3=3) (4=2) (5=1), generate(CULTURE_11_6_reverse_code)

egen individualism_scale = rmean(CULTURE_11_1 CULTURE_11_2_reverse_code CULTURE_11_3 CULTURE_11_4 CULTURE_11_5_reverse_code CULTURE_11_6_reverse_code)

recode individualism_scale (1/1.9=1) (2/2.9=2) (3/3.9=3) (4/4.9=4) (5=5), generate(individualism_scale_ord)

recode individualism_scale_ord (4 5=4)

recode individualism_scale_ord (4=1) (3=2) (2=3) (1=4)

# SUPPLEMENT 2: EXPERIMENTAL ANALYSIS, BALANCE TABLE

#### **S2 Table 1: Randomization- Balance across Study Arms**

|  | Control Arm - Broader Risk  (%) | Experimental Arm - Racial Disparities  (%) | Total  (%) | p-value |
| --- | --- | --- | --- | --- |
| High school | 181 | 206 | 387 |  |
|  | 26.97 | 30.34 | 28.67 |  |
| Some college | 181 | 183 | 364 |  |
|  | 26.97 | 26.95 | 26.96 |  |
| College | 173 | 161 | 334 |  |
|  | 25.78 | 23.71 | 24.74 |  |
| Graduate degree+ | 136 | 129 | 265 |  |
|  | 20.27 | 19 | 19.63 | 0.533 |
| Male | 330 | 337 | 667 |  |
|  | 49.03 | 49.56 | 49.3 |  |
| Female | 343 | 343 | 686 |  |
|  | 50.97 | 50.44 | 50.7 | 0.847 |
| 18-29 | 200 | 218 | 418 |  |
|  | 29.72 | 32.06 | 30.89 |  |
| 30-39 | 182 | 159 | 341 |  |
|  | 27.04 | 23.38 | 25.2 |  |
| 40-49 | 106 | 98 | 204 |  |
|  | 15.75 | 14.41 | 15.08 |  |
| 50-59 | 89 | 84 | 173 |  |
|  | 13.22 | 12.35 | 12.79 |  |
| >60 | 96 | 121 | 217 |  |
|  | 14.26 | 17.79 | 16.04 | 0.229 |
| <$50,000 | 296 | 305 | 601 |  |
|  | 43.98 | 44.85 | 44.42 |  |
| $50,000-$100,000 | 122 | 127 | 249 |  |
|  | 18.13 | 18.68 | 18.4 |  |
| >$100,000 | 255 | 248 | 503 |  |
|  | 37.89 | 36.47 | 37.18 | 0.862 |
| Vote Biden | 400 | 425 | 825 |  |
|  | 48.48 | 51.52 | 100.00 |  |
| Vote Trump | 99 | 95 | 194 |  |
|  | 51.03 | 48.97 | 100.00 |  |
| Didn't vote/vote other | 174 | 160 | 334 |  |
|  | 52.10 | 47.90 | 100.00 | 0.499 |
| NH White | 203 | 226 | 429 |  |
|  | 30.16 | 33.24 | 31.71 |  |
| NH Black | 226 | 217 | 443 |  |
|  | 33.58 | 31.91 | 32.74 |  |
| Hispanic | 244 | 237 | 481 |  |
|  | 36.26 | 34.85 | 35.55 | 0.477 |
| Total | 673 | 680 | 1,353 |  |
|  | 100 | 100 | 100 |  |

**S2 Table 2: Race and Ethnicity by Vote Choice**

|  | Hispanic | NH Black | NH White | Total |
| --- | --- | --- | --- | --- |
| Vote Biden | 262 | 300 | 263 | 825 |
|  | 58.48 | 67.72 | 56.93 | 60.98 |
| Vote Trump | 55 | 22 | 117 | 194 |
|  | 12.28 | 4.97 | 25.32 | 14.34 |
| Did not vote/vote other | 131 | 121 | 82 | 334 |
|  | 29.24 | 27.31 | 17.75 | 24.69 |
| Total | 448 | 443 | 462 | 1,353 |
|  | 100 | 100 | 100 | 100 |

# SUPPLEMENT 3: QUANTITATIVE ROBUSTNESS CHECKS

*Robustness Checks*. The results of the robustness checks with logistic regression and party identification were mostly consistent with results from ologit models with vote choice with a few caveats. In the stepwise logistic regression models, the introduction of the effect mediators reduced the strength of association between vote choice and hesitancy, but did eliminate the signifacne voting for Trump on hesitancy compared with Biden voters even after the introduction of these measures (Table A3.1.). In logistic models, the experiment showed some evidence of heterogenous effects by vote choice. Specifically, Biden voters exposed to the disparities conditions were more hesitant compared with Biden voters in the control condition and non/other voters in the experimental condition were less hesitant than their counterparts in the control condition (Table A3.3.). We do not emphasize these results as they do not hold up in the ologit models, nor in the results by party identification as discussed more below. Ologit models with party identification found that, contrary to other findings, Independents rather than Republicans were more hesitant compared with Democrats. This effect diminished but not disappear with the introduction of mediating variables (Table A3.4.). There was no interaction effect between the experiment and party identification in either ologit or logistic models (Table A3.5. & Table A3.6.).

**S3 Table 1: Logistic Stepwise**

|  | No controls | Demographic Controls | Controls and Confounders | Controls, Confounders and Mediators |
| --- | --- | --- | --- | --- |
| VARIABLES | odds ratio | odds ratio | odds ratio | odds ratio |
| EXP1 | 1 | 1.01 | 0.99 | 0.97 |
|  | (0.737 - 1.370) | (0.735 - 1.397) | (0.718 - 1.371) | (0.697 - 1.353) |
| Race/Ethnicity |  |  |  |  |
| NH White (ref) | ref | ref | ref | ref |
| NH Black | 1.74*** | 1.58** | 1.63** | 1.82*** |
|  | (1.179 - 2.579) | (1.041 - 2.401) | (1.065 - 2.491) | (1.161 - 2.849) |
| Hispanic | 1.06 | 0.97 | 1.06 | 1.14 |
|  | (0.706 - 1.594) | (0.627 - 1.494) | (0.683 - 1.647) | (0.724 - 1.805) |
| Vote Choice 2020 |  |  |  |  |
| Vote Biden 2020 | ref | ref | ref | ref |
| Vote Trump 2020 | 2.07*** | 3.01*** | 2.98*** | 1.94** |
|  | (1.336 - 3.218) | (1.873 - 4.849) | (1.843 - 4.815) | (1.154 - 3.275) |
| Did not vote 2020 | 1.67*** | 1.70*** | 1.78*** | 1.37 |
|  | (1.170 - 2.387) | (1.157 - 2.494) | (1.208 - 2.635) | (0.915 - 2.064) |
| Female |  | 2.48*** | 2.40*** | 2.60*** |
|  |  | (1.738 - 3.545) | (1.678 - 3.445) | (1.792 - 3.767) |
| Age |  |  |  |  |
| 18-30 (ref) |  | ref | ref | ref |
| 30-39 |  | 1.17 | 1.24 | 1.4 |
|  |  | (0.736 - 1.855) | (0.778 - 1.980) | (0.865 - 2.261) |
| 40-49 |  | 1.71** | 1.79** | 2.13*** |
|  |  | (1.023 - 2.859) | (1.064 - 3.013) | (1.244 - 3.640) |
| 50-59 |  | 2.06*** | 2.09*** | 2.39*** |
|  |  | (1.254 - 3.397) | (1.259 - 3.462) | (1.424 - 4.028) |
| 60+ |  | 1.31 | 1.45 | 1.94** |
|  |  | (0.763 - 2.255) | (0.832 - 2.534) | (1.082 - 3.463) |
| Education |  |  |  |  |
| < College (ref) |  | ref | ref | ref |
| Some College |  | 1.53** | 1.53** | 1.52* |
|  |  | (1.010 - 2.306) | (1.011 - 2.330) | (0.990 - 2.326) |
| College |  | 1.04 | 1.01 | 1 |
|  |  | (0.648 - 1.674) | (0.622 - 1.626) | (0.609 - 1.633) |
| Graduate Degree |  | 0.87 | 0.9 | 0.93 |
|  |  | (0.490 - 1.556) | (0.501 - 1.607) | (0.516 - 1.683) |
| Income |  |  |  |  |
| <$50,000 (ref) |  | ref | ref | ref |
| $50,000-$100,000 |  | 0.62** | 0.63** | 0.7 |
|  |  | (0.390 - 0.976) | (0.396 - 0.997) | (0.437 - 1.126) |
| >$100,000 |  | 0.44*** | 0.45*** | 0.55*** |
|  |  | (0.289 - 0.683) | (0.294 - 0.702) | (0.351 - 0.856) |
| Co-morbidities |  |  |  |  |
| 0 (ref) |  |  | ref | ref |
| 1 |  |  | 0.83 | 0.85 |
|  |  |  | (0.579 - 1.199) | (0.587 - 1.238) |
| 2 |  |  | 0.65 | 0.67 |
|  |  |  | (0.371 - 1.124) | (0.381 - 1.179) |
| 3+ |  |  | 0.53* | 0.49* |
|  |  |  | (0.256 - 1.087) | (0.230 - 1.022) |
| Family die COVID-19 |  |  | 0.50** | 0.50** |
|  |  |  | (0.278 - 0.887) | (0.276 - 0.904) |
| Religion Very Important |  |  |  | 0.94 |
|  |  |  |  | (0.661 - 1.350) |
| Alternative Media |  |  |  | 1.97*** |
|  |  |  |  | (1.343 - 2.897) |
| Trust in Medico-Pharm Institutions |  |  |  |  |
| Complete (ref) |  |  |  | ref |
| A great deal |  |  |  | 1.3 |
|  |  |  |  | (0.777 - 2.175) |
| Some |  |  |  | 1.72** |
|  |  |  |  | (1.017 - 2.901) |
| Very little |  |  |  | 3.53*** |
|  |  |  |  | (1.889 - 6.595) |
| Individualism score (high) |  |  |  | 1.46** |
|  |  |  |  | (1.034 - 2.063) |
| Constant | 0.10*** | 0.06*** | 0.07*** | 0.03*** |
|  | (0.069 - 0.146) | (0.029 - 0.105) | (0.034 - 0.128) | (0.012 - 0.060) |
| Observations | 1,353 | 1,350 | 1,348 | 1,347 |
| *** p<0.01, ** p<0.05, * p<0.1 |  |  |  |  |

Notes: DV= Definitely will not vaccinate (1), Else (0)

**S3 Table 2. Ologit, Stepwise with Party Identification**

|  | No controls | Demographic Controls | Controls and Confounders | Controls, Confounders and Mediators |
| --- | --- | --- | --- | --- |
| VARIABLES | odds ratio | odds ratio | odds ratio | odds ratio |
| EXP1 | 1.04 | 1.03 | 1.03 | 0.99 |
|  | (0.857 - 1.270) | (0.846 - 1.264) | (0.843 - 1.262) | (0.803 - 1.212) |
| Race/Ethnicity |  |  |  |  |
| NH White (ref) | ref | ref | ref | ref |
| NH Black | 1.98*** | 1.79*** | 1.82*** | 1.99*** |
|  | (1.547 - 2.540) | (1.376 - 2.330) | (1.399 - 2.379) | (1.512 - 2.628) |
| Hispanic | 1.35** | 1.25* | 1.37** | 1.43** |
|  | (1.057 - 1.721) | (0.966 - 1.629) | (1.055 - 1.792) | (1.088 - 1.872) |
| Vote Choice 2020 |  |  |  |  |
| Democrat (ref) | ref | ref | ref | ref |
| Republican | 0.9 | 1.07 | 1.09 | 0.87 |
|  | (0.692 - 1.173) | (0.816 - 1.404) | (0.829 - 1.436) | (0.655 - 1.155) |
| Independent | 2.22*** | 2.33*** | 2.18*** | 1.66*** |
|  | (1.743 - 2.824) | (1.820 - 2.994) | (1.694 - 2.799) | (1.282 - 2.150) |
| Female |  | 1.82*** | 1.76*** | 1.94*** |
|  |  | (1.475 - 2.245) | (1.424 - 2.176) | (1.566 - 2.413) |
| Age |  |  |  |  |
| 18-30 (ref) |  | ref | ref | ref |
| 30-39 |  | 1.01 | 1.05 | 1.27 |
|  |  | (0.762 - 1.330) | (0.794 - 1.392) | (0.952 - 1.696) |
| 40-49 |  | 1.18 | 1.23 | 1.58*** |
|  |  | (0.850 - 1.645) | (0.880 - 1.711) | (1.120 - 2.222) |
| 50-59 |  | 1.72*** | 1.63*** | 1.97*** |
|  |  | (1.228 - 2.420) | (1.159 - 2.304) | (1.392 - 2.780) |
| 60+ |  | 1.22 | 1.23 | 1.76*** |
|  |  | (0.888 - 1.689) | (0.885 - 1.715) | (1.246 - 2.479) |
| Education |  |  |  |  |
| < College (ref) |  | ref | ref | ref |
| Some College |  | 1.72*** | 1.69*** | 1.73*** |
|  |  | (1.304 - 2.264) | (1.284 - 2.237) | (1.303 - 2.285) |
| College |  | 1.25 | 1.19 | 1.26 |
|  |  | (0.936 - 1.678) | (0.886 - 1.596) | (0.937 - 1.702) |
| Graduate Degree |  | 0.75* | 0.73* | 0.81 |
|  |  | (0.533 - 1.048) | (0.519 - 1.025) | (0.575 - 1.152) |
| Income |  |  |  |  |
| <$50,000 (ref) |  | ref | ref | ref |
| $50,000-$100,000 |  | 0.71** | 0.71** | 0.8 |
|  |  | (0.529 - 0.940) | (0.533 - 0.951) | (0.595 - 1.069) |
| >$100,000 |  | 0.61*** | 0.63*** | 0.70*** |
|  |  | (0.475 - 0.794) | (0.483 - 0.809) | (0.540 - 0.911) |
| Co-morbidities |  |  |  |  |
| 0 (ref) |  |  | ref | ref |
| 1 |  |  | 0.83 | 0.82 |
|  |  |  | (0.658 - 1.053) | (0.648 - 1.047) |
| 2 |  |  | 0.83 | 0.81 |
|  |  |  | (0.602 - 1.141) | (0.587 - 1.125) |
| 3+ |  |  | 0.67* | 0.61** |
|  |  |  | (0.448 - 1.012) | (0.399 - 0.921) |
| Family die COVID-19 |  |  | 0.49*** | 0.51*** |
|  |  |  | (0.364 - 0.663) | (0.372 - 0.693) |
| Religion Very Important |  |  |  | 0.67*** |
|  |  |  |  | (0.533 - 0.833) |
| Alternative Media |  |  |  | 1.47*** |
|  |  |  |  | (1.123 - 1.918) |
| Trust in Medico-Pharm Institutions |  |  |  |  |
| Complete (ref) |  |  |  | ref |
| A great deal |  |  |  | 2.06*** |
|  |  |  |  | (1.532 - 2.766) |
| Some |  |  |  | 3.19*** |
|  |  |  |  | (2.333 - 4.366) |
| Very little |  |  |  | 3.87*** |
|  |  |  |  | (2.527 - 5.924) |
| Individualism score (high) |  |  |  | 1.45*** |
|  |  |  |  | (1.168 - 1.796) |
| /cut1 | 1.2 | 1.59** | 1.24 | 3.40*** |
|  | (0.953 - 1.519) | (1.080 - 2.336) | (0.825 - 1.871) | (2.088 - 5.531) |
| /cut2 | 3.82*** | 5.46*** | 4.39*** | 13.01*** |
|  | (2.989 - 4.872) | (3.678 - 8.114) | (2.892 - 6.655) | (7.876 - 21.496) |
| /cut3 | 11.04*** | 16.75*** | 13.67*** | 42.87*** |
|  | (8.434 - 14.450) | (11.055 - 25.380) | (8.853 - 21.116) | (25.387 - 72.390) |
| Observations | 1,353 | 1,350 | 1,348 | 1,347 |
| *** p<0.01, ** p<0.05, * p<0.1 | |  |  |  |

**S3 Table 4: Summary of Attention Check Responses to Experimental Condition**

|  | N | % |
| --- | --- | --- |
| High-risk healthcare workers and first responders | 441 | 64.9 |
| High-risk healthcare workers and first responders & Low-income Minorities | 81 | 11.9 |
| High-risk healthcare workers and first responders, Low-income Minorities & Healthy children and young adults | 37 | 5.4 |
| High-risk healthcare workers and first responders & Healthy children and young adults | 22 | 3.2 |
| Low-income Minorities | 97 | 14.3 |
| Low-income Minorities & Healthy children and young adults | 2 | 0.29 |
| Total | 680 | 100 |
| **Control Condition** | **673** |  |

**S3 Table 5: Experiment Interacted w/Race & Ethnicity, respondents failing attention check excluded**

| VARIABLES+A3:B45 | odds ratio |
| --- | --- |
| Hispanic (ref) | ref |
| NH White | 0.99 |
|  | (0.684 - 1.432) |
| NH Black | 1.55** |
|  | (1.109 - 2.180) |
| Experimental Arm | 1.27 |
|  | (0.898 - 1.809) |
| NH White#Experimental Arm | 0.76 |
|  | (0.458 - 1.261) |
| NH Black#Experimental Arm | 0.7 |
|  | (0.428 - 1.141) |
| Observations | 1,290 |
| NOTES: |  |
| ciEform in parentheses |  |
| *** p<0.01, ** p<0.05, * p<0.1 |  |
| Gender, Income, Education and Age included but not shown | |
|  |  |

**S3 Table 6: Experiment Interacted w/Vote Choice, respondents failing attention check excluded**

| VARIABLES | odds ratio |
| --- | --- |
| Vote Choice Biden (2020) (ref) | ref |
| Vote Choice Trump 2020 | 1.99*** |
|  | (1.299 - 3.048) |
| Vote Choice Other/No Vote 2020 | 1.65*** |
|  | (1.176 - 2.312) |
| Experimental Arm | 1.2 |
|  | (0.920 - 1.552) |
| Vote Trump#Experiment | 0.64 |
|  | (0.348 - 1.183) |
| Vote Other#Experiment | 0.72 |
|  | (0.443 - 1.158) |
| Observations | 1,290 |
| Notes: Ordered Logistic Regression; *** p<0.01, ** p<0.05, * p<0.1  ci Eform in parentheses; Gender, Income, Education and Age included but not shown | |

# SUPPLEMENT 4: QUALITATIVE ANALYSIS

All qualitative analyses were conducted in Excel. We conducted first-round coding inductively, retaining verbatim terms where possible. Each response could be assigned more than one code when the respondent expressed multiple ideas in their response. First-round coding was further refined and aggregated, yielding 65 inductive codes.

To connect our analysis to the broader literature, we then deductively fit these codes into a modified ‘5Cs’ framework of the psychological antecedents of vaccine preferences (Confidence, Complacency, Constraints, Calculation, Collective Responsibility (Betsch et al., 2018; MacDonald et al., 2015). The 5Cs build on several previous frameworks of vaccine attitudes and was appealing both because of (a) more categories, and therefore nuance, than earlier frameworks and (b) its specific application in a high-income country. The framework introduces five key categories (Betsch et al., 2018):

- Confidence: This includes three dimensions of trust — “(i) the effectiveness and safety of vaccines, (ii) the system that delivers them, (iii) the motivations of policy-makers who decide on the need for vaccines.”
- Complacency(concern): This includes two dimensions: (i) perceived risk of vaccine-preventable disease and (ii) assessment of vaccine as a necessary preventative action.
- Constraints: This includes multiple possible dimensions: (i) physical availability or supply, (ii) affordability and willingness-to-pay, (iii) geographic accessibility, (iv) ability to understand (language and health literacy), (v) appeal of immunization service, and (vi) opportunity costs or time pressures.
- Calculation: This relates to engagement in information-seeking in order to make a vaccination decision.
- Collective responsibility: “willingness to protect others by one’s own vaccination” by means of herd immunity.

We modified the framework in two ways: first, to more easily use the framework to represent reasons *both* acceptance and hesitancy of vaccinations in line with out research interests and, second, deductively from the data, to add a category to those codes we found specific to the pandemic context.

This yielded six parent codes and forty-four child codes. All authors reviewed and commented on these codes and categorization and suggestion revisions, which were then incorporated into a codebook. One author then used this codebook to re-code all the original data, iteratively refining the codebook was needed. Two more authors reviewed this coding, adjusting and adding as needed to be sure we captured relevant nuance. Differences were discussed and reconciled for a final codebook and re-coding of the data for further analysis.

#### **S4 Table 1. Stated reasons for wanting/not wanting to get the vaccine by race-ethnicity (major emergent categories)**

| Statements about Vaccine Intentions | NH White + Other (%) | NH Black (%) | Hispanic (%) | Chi2, p-value |
| --- | --- | --- | --- | --- |
| Positive Statements |  |  |  |  |
| Trust in Vaccine(s) | 2.08 | 2.55 | 2.51 | 0.910 |
| Trust in System - High | 3.56 | 2.55 | 2.19 | 0.545 |
| Trust in Motivation of Policymakers - High | 0.30 | 1.82 | 1.57 | 0.164 |
| Perceived Individual Risks of COVID - High | 21.96 | 14.91 | 12.85 | 0.005 |
| Perceived Value of Vaccine for Self/Family - High | 45.70 | 42.91 | 43.89 | 0.777 |
| Collective Responsibility | 15.73 | 10.18 | 9.40 | 0.025 |
| Pandemic specific: To Resume Normal Life | 10.98 | 8.36 | 7.84 | 9.130 |
| Negative Statements |  |  |  |  |
| Distrust in Vaccine(s) | 57.60 | 47.02 | 56.59 | 0.126 |
| Trust in System - Low | 15.20 | 11.90 | 9.30 | 0.352 |
| Trust in Motivation of Policymakers - Low | 8.00 | 9.52 | 2.33 | 0.044 |
| Conspiracy Theories | 4.00 | 4.17 | 0.78 | 0.191 |
| Desire to Wait & See | 35.20 | 25.60 | 31.78 | 0.191 |
| Constraints (Supply, Access, etc.) | 5.60 | 3.57 | 3.10 | 0.555 |

#### **S4 Table 2. Stated reasons for wanting/not wanting to get the vaccine by party identity (major emergent categories)**

| Statements about Vaccine Intentions | Democrat (%) | Republican (%) | Independent (%) | Chi2, p-value |
| --- | --- | --- | --- | --- |
| Positive Statements |  |  |  |  |
| Trust in Vaccine(s) | 1.28 | 4.81 | 2.84 | 0.015 |
| Trust in System - High | 2.74 | 1.44 | 4.55 | 0.183 |
| Trust in Motivation of Policymakers - High | 1.28 | 1.44 | 0.57 | 0.693 |
| Perceived Individual Risks of COVID - High | 15.72 | 13.94 | 23.30 | 0.030 |
| Perceived Value of Vaccine for Self/Family - High | 45.52 | 38.94 | 46.59 | 0.210 |
| Collective Responsibility | 12.80 | 11.06 | 10.23 | 0.598 |
| Pandemic specific: To Resume Normal Life | 8.59 | 8.17 | 11.93 | 0.352 |
| Negative Statements |  |  |  |  |
| Distrust in Vaccine(s) | 53.92 | 46.77 | 54.55 | 0.556 |
| Trust in System - Low | 12.44 | 14.52 | 10.49 | 0.700 |
| Trust in Motivation of Policymakers - Low | 5.99 | 6.45 | 8.39 | 0.672 |
| Conspiracy Theories | 2.30 | 4.84 | 3.50 | 0.559 |
| Desire to Wait & See | 32.72 | 35.48 | 24.48 | 0.158 |
| Constraints (Supply, Access, etc.) | 4.15 | 3.23 | 4.20 | 0.941 |

# SUPPLEMENT 5: STUDY ARMS AND QUESTIONNAIRE/ QUESTION WORDING*

*Note: This is an abridged version of the questionnaire that included question relevant to the present study

### Consent Question:

This research is being conducted by XXX. It is a study of public opinion on issues relating to health care.

Your participation is voluntary. Participation involves taking an online survey that lasts approximately 15 minutes. You may choose to not answer any or all questions.

The information collected will be maintained anonymously. Names and other identifying information will not be used in any presentation or paper written about this project.

Questions about this project may be directed to: XXX.

Do you consent to participate in this survey?

- Yes, I consent
- No, I do not

*This will be the only forced-response question in the survey. If the respondent does not give his or consent, they will be thanked for their time and the survey will end.*

### Newspaper Article

*The respondent will be shown one of the following 2 versions of the newspaper text or a control condition [no newspaper text] (selected at random):*

Please take a few minutes to carefully read the following newspaper article.  Once you are finished, you will be asked a few questions to better understand your perspective on the article and gauge your opinions on current events.

T1: Racial Disparities Condition (Control)


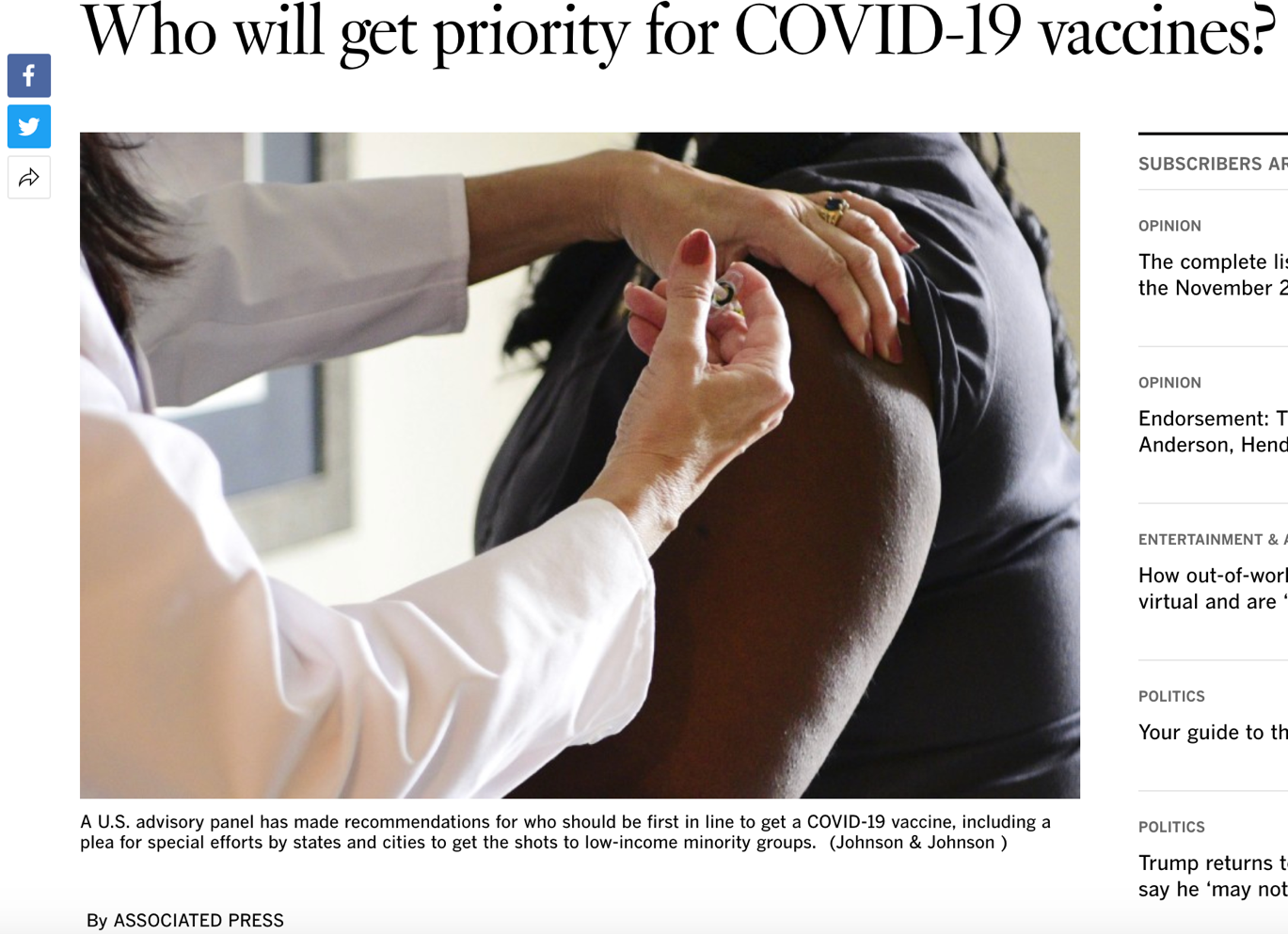


A U.S. advisory panel made recommendations Friday for [who should be first in line to get doses of COVID-19 vaccine](https://www.nap.edu/catalog/25917/framework-for-equitable-allocation-of-covid-19-vaccine), including a plea for special efforts by states and cities to get the shots to low-income minority groups.

As expected, the panel recommended healthcare workers and first responders get priority when vaccine supplies are limited. The shots should be provided free to all, the panel said. And throughout the vaccine campaign, efforts also should focus on disadvantaged areas to remedy racial health disparities, according to the report from the National Academies of Sciences, Engineering and Medicine.

“Everybody knows from the news how deadly this has been for minorities,” said [Dr. William Foege](http://www.ph-leader.emory.edu/people/bio-section-faculty/foege-william.html) of Emory Rollins School of Public Health, co-chair of the panel. “We said it’s racism that is the root cause of this problem.

“This virus has no concept of color, but it has a very good concept of vulnerabilities,” he added.

### Comprehension Questions

C1_Q1: Who did U.S. advisory panel recommend get the vaccine *first* while supplies are scarce [check all that apply]?

- High-risk healthcare workers and first responders
- Low-income Minorities
- Healthy children and young adults

C1_Q3: Did the U.S. advisory panel recommend the vaccine be free?

1. Yes
2. No

[Red responses= accept as correct]

T2: Broader Risk Group (White picture, Elderly & Health Workers) Frame


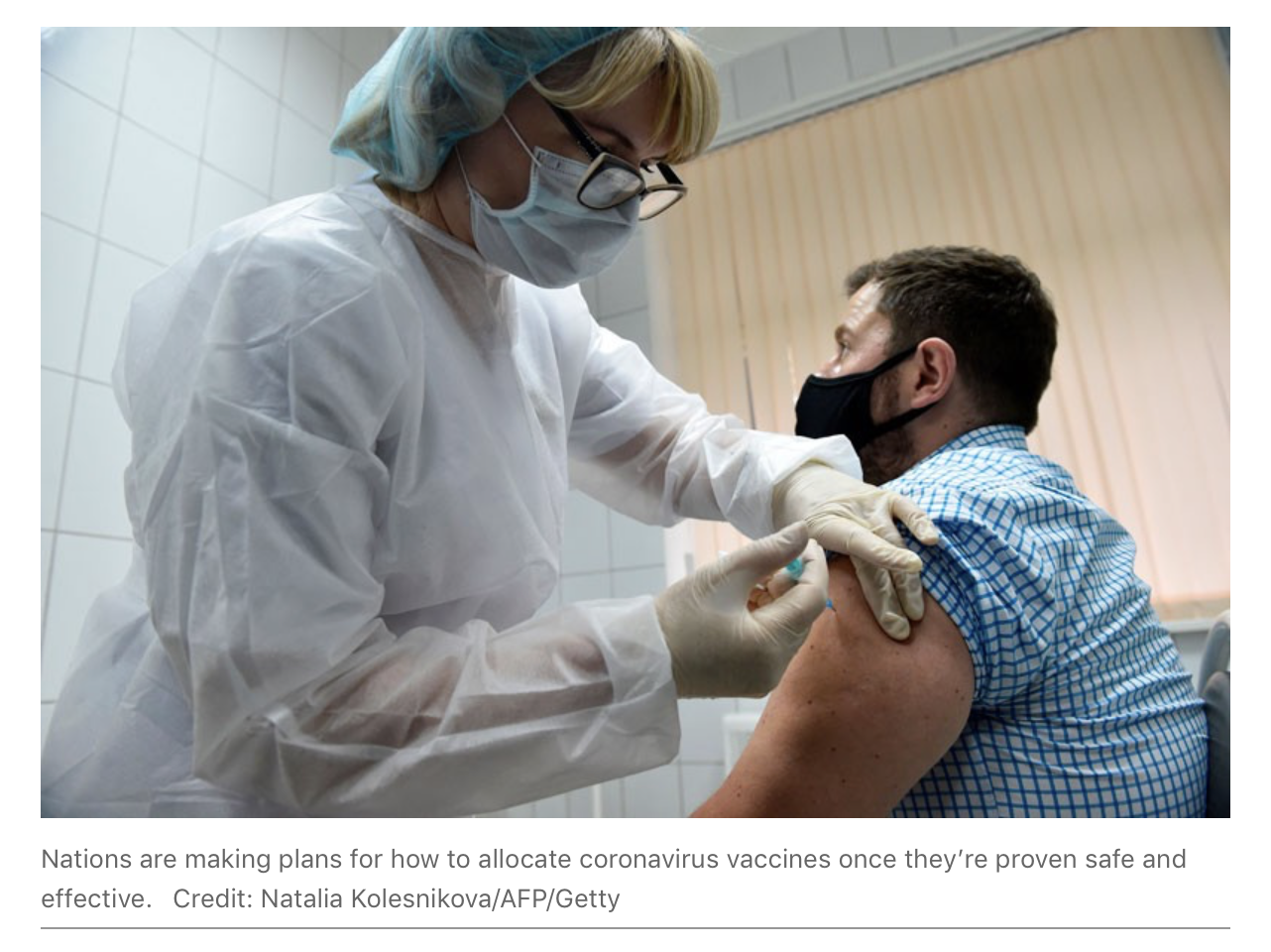

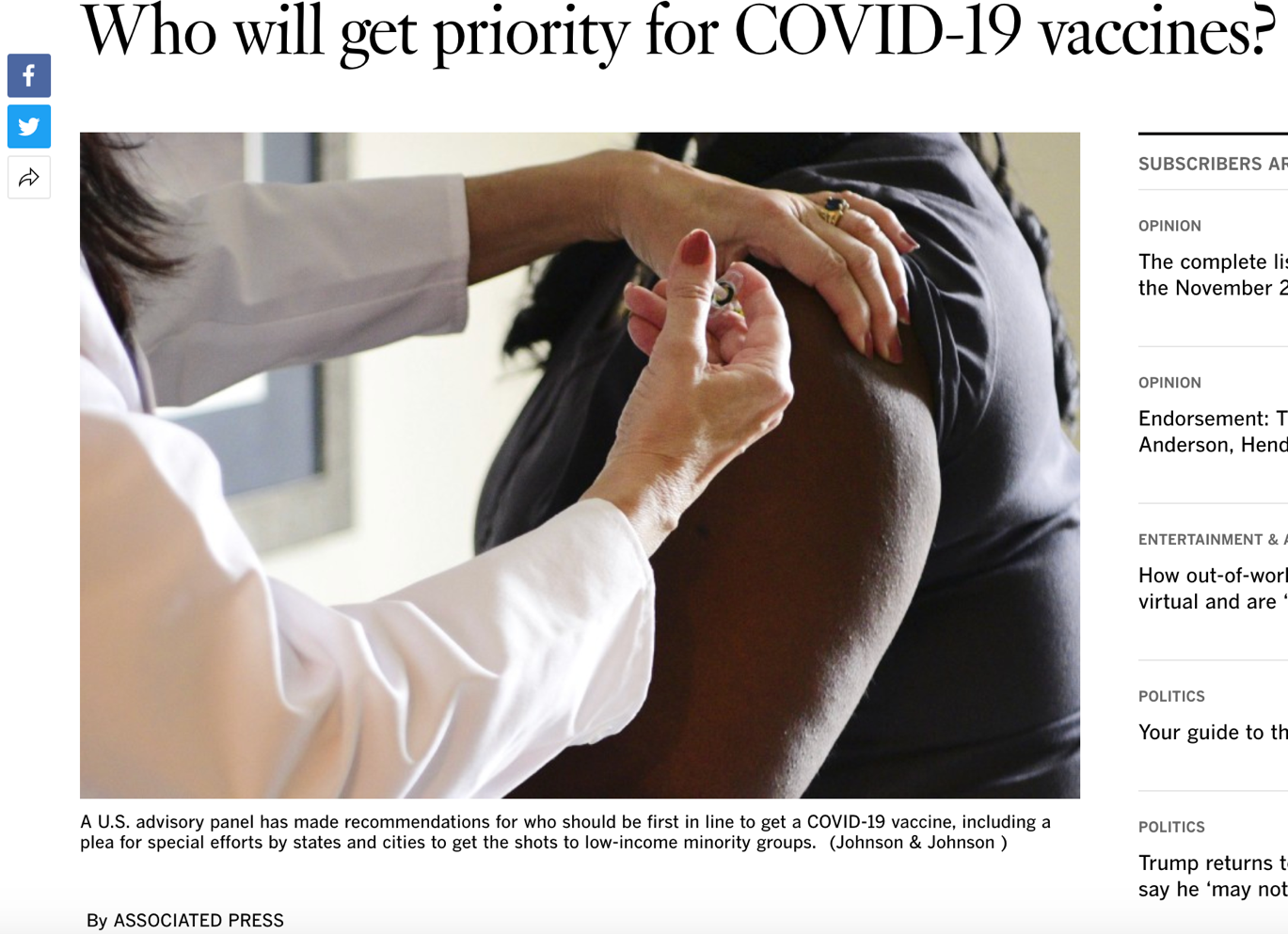


A U.S. advisory panel made recommendations Friday for [who should be first in line to get doses of COVID-19 vaccine](https://www.nap.edu/catalog/25917/framework-for-equitable-allocation-of-covid-19-vaccine). The panel recommended healthcare workers and first responders get priority when vaccine supplies are limited. The shots should be provided free to all, the panel said.

The National Academies document lays out successive waves of vaccine distribution as manufacturing ramps up:

- When supplies are scarce, the first doses should go to high-risk healthcare workers in hospitals, nursing homes and those providing home care. First responders also would be in this group.
- Next, older residents of nursing homes and other crowded facilities and people of all ages with health conditions that put them at significant danger. The report said it remains unclear which health conditions should be included. It lists cancer, chronic kidney disease and obesity among possibilities.
- In following waves, teachers, child care workers, workers in essential industries — specific job categories might vary by state — and people living in homeless shelters, group homes, prisons and other facilities.
- Once supplies increase, healthy children, young adults and everyone else.

### Comprehension Questions

C2_Q1: Who did U.S. advisory panel recommend get the vaccine first while supplies are scarce?

1. High-risk healthcare workers
2. Older residents of nursing homes
3. Teachers and childcare workers
4. Healthy children and young adults

C2_Q2: Did the U.S. advisory panel recommend the vaccine be free?

1. Yes
2. No

[Red responses= accept as correct]

### Questionnaire on Attitudes towards Vaccination

The following set of questions will ask you about your attitudes towards the COVID-19 vaccine and COVID-19 prevention measures.

Q1. [from [Pew](https://www.pewresearch.org/science/wp-content/uploads/sites/16/2020/09/PS_2020.09.17_COVID-19-Vaccine_TOPLINE.pdf)]. If a vaccine to prevent COVID-19 was approved by the FDA through normal procedures and available today for free to the public, would you...

1. Definitely GET the vaccine as soon as possible
2. Probably GET the vaccine as soon as possible
3. Probably NOT GET the vaccine as soon as possible
4. Definitely NOT GET the vaccine as soon as possible

Q1A. Briefly describe why you would get the vaccine as soon as possible___________________________________________________

Q1B. Briefly describe why you would not get the vaccine as soon as possible___________________________________________________

Q2. [from [Pew](https://www.pewresearch.org/science/wp-content/uploads/sites/16/2020/09/PS_2020.09.17_COVID-19-Vaccine_TOPLINE.pdf)]. Regardless of how likely you are to get the COVID-19 vaccine if it becomes available, how much of a reason, if any, is each of the following for why you would NOT get a vaccine to prevent COVID-19? **[RANDOMIZE]**

|  | A major reason | A minor reason | Not a reason |
| --- | --- | --- | --- |
| a. Concern about side effects |  |  |  |
| b. Do not think I need it because the disease is not that bad |  |  |  |
| c. Do not think I need it because I am not in a high-risk group |  |  |  |
| d. Want to know more about how well it works |  |  |  |
| e. Afraid of needles |  |  |  |
| f. Religious objection |  |  |  |

Q3. Which of the following best describes your own belief about the seriousness of Coronavirus as a threat to the nation’s health and the need for renewed social distancing measures?

1. This is all an overreaction. The disease is not that serious. We should relax social distancing measures right away and not introduce new ones.
2. Coronavirus is still a serious threat and we should re-introduce some social distancing measures in order to reduce the spread of the virus, but not go back into full on lock-down.
3. The disease is a large threat to the health of the nation that will force unconscionable choices if we do not all act together to socially distance for as long as needed even if that means great personal sacrifice.

Q4. Which of the following statements comes closer to your view:

1. While Coronavirus is a serious illness, the threat to the economy is a bigger threat. We need to prioritize opening up the economy and putting people back to work.
2. People should not put economic concerns above people’s health. We will need to socially distance as long as it takes to reduce the health risk.

Q5. How frequently have you been doing each of the following over the past month to personally protect yourself and others from Coronavirus?

|  | All of the time | Most of the time | Some of the Time | Not at All | NA |
| --- | --- | --- | --- | --- | --- |
| 1. Worn a mask when out in public |  |  |  |  |  |
| 1. Washed hands after being out |  |  |  |  |  |
| 1. Used hand sanitizer at stores or when out |  |  |  |  |  |
| 1. Sought out testing for Coronavirus before seeing someone who may be vulnerable to complications |  |  |  |  |  |
| 1. Changed travel plans or decided not to travel |  |  |  |  |  |
| 1. Avoided contact with people who who may be vulnerable to complications (e.g., grandparents, immunocompromised, etc) |  |  |  |  |  |
| 1. Avoided large or medium size gatherings |  |  |  |  |  |
| 1. Avoided small group gatherings |  |  |  |  |  |
| 1. Sought information on Coronavirus |  |  |  |  |  |
| 1. Self-quarantined/isolated |  |  |  |  |  |

When it comes to responding to the Coronavirus, do you mostly support or oppose requiring people to wear face masks indoors and where social distancing isn’t possible.

a. Strongly Support

b. Somewhat Support

c. Somewhat Oppose

d. Strongly Oppose

Does wearing a mask help to reduce the spread of the coronavirus?

a. Yes, a lot

b. Yes, some

c. Not sure

d. No, it does nothing

e. No, it increases the spread

Q5. [from [Pew](https://www.pewresearch.org/science/wp-content/uploads/sites/16/2020/09/PS_2020.09.17_COVID-19-Vaccine_TOPLINE.pdf)]. How much confidence, if any, do you have that the research and development process will produce a vaccine for COVID-19 in the U.S. that is safe and effective in the next few months?

1. A great deal of confidence
2. A fair amount of confidence
3. Not too much confidence
4. No confidence at all

Q6. [from [Pew](https://www.pewresearch.org/science/wp-content/uploads/sites/16/2020/09/PS_2020.09.17_COVID-19-Vaccine_TOPLINE.pdf)]. Thinking about the development of a vaccine for COVID-19, which of the following concerns you more?

1. It is developing too fast without fully establishing it is safe and effective
2. It is developing too slowly, creating unnecessary delays in providing access to a vaccine
3. No concerns, it is developing at the right pace

Q8. Please indicate how much you disagree or agree with the following statements about a COVID- 19 vaccine [randomize].

|  | Strongly agree | Somewhat agree | Somewhat disagree | Strongly Disagree |
| --- | --- | --- | --- | --- |
| 1. Getting a COVID-19 vaccine myself will be an important way **to protect myself** from catching the disease |  |  |  |  |
| 1. Getting a COVID-19 vaccine myself will be an important way to protect **my immediate family members and friends** from catching the disease |  |  |  |  |
| 1. Getting a COVID-19 vaccine myself will be an important way to protect **vulnerable members of my community** from catching the disease |  |  |  |  |
| 1. Everyone who doesn’t have a medical reason not to should get a COVID-19 vaccine to help end the pandemic |  |  |  |  |
| 1. For a future COVID-19 vaccine, the health benefits of getting the vaccine are likely to outweigh the risks |  |  |  |  |

Q9. Please indicate how much you disagree or agree with the following statements about vaccines in general (for all diseases) [randomize].

|  | Strongly agree | Somewhat agree | Somewhat disagree | Strongly Disagree |
| --- | --- | --- | --- | --- |
| 1. The side effects for most vaccines are minor |  |  |  |  |
| 1. Serious side effects from vaccines are rare |  |  |  |  |
| 1. You can catch the flu from a flu vaccine |  |  |  |  |
| 1. When everyone else is vaccinated, I don't need to be vaccinated* |  |  |  |  |
| 1. I have a responsibility to get vaccinated because I can protect others with a weaker immune system* |  |  |  |  |
| 1. When I think about getting vaccinated, I weigh benefits and risks to make the best decision possible* |  |  |  |  |
| 1. Vaccines contain dangerous ingredients |  |  |  |  |
| 1. The decision to vaccinate or not is a personal choice |  |  |  |  |

***Modifications from the** vaccine hesitancy scale:

<https://www.ncbi.nlm.nih.gov/pmc/articles/PMC6285469/table/pone.0208601.t005/?report=objectonly>

Q11. How much confidence, if any, do you have that each of the following will act in the best interest of the public when it comes to researching, developing, and distributing a COVID-19 vaccine as of today?

|  | No confidence at all | Very little confidence | Some confidence | A great deal of confidence | Complete confidence |
| --- | --- | --- | --- | --- | --- |
| a. Elected federal government officials under the Biden Administration |  |  |  |  |  |
| b. Federal health agencies (e.g., the FDA and CDC) |  |  |  |  |  |
| b. l public health agencies |  |  |  |  |  |
| c. Medical scientists and researchers |  |  |  |  |  |
| d. Physicians |  |  |  |  |  |
| e. U.S.-based Pharmaceutical  companies |  |  |  |  |  |
| f. Chinese-based researchers/companies |  |  |  |  |  |
| g. European-based researchers/companies |  |  |  |  |  |

Q12. How often do you typically get a seasonal flu vaccination?

1. Never
2. Some years
3. Every year

Q13. Since August 2020, have you had a seasonal flu vaccination?

1. Yes
2. No
3. Don’t remember

### Vaccine Delivery

Once a COVID1-9 vaccine is widely available, how much would the following influence your decision to get the vaccine:

|  | Much more likely | Somewhat more likely | Somewhat less likely | Much less likely | No effect or Not Applicable |
| --- | --- | --- | --- | --- | --- |
| If it is required in order to board a plane or go on a cruise |  |  |  |  |  |
| If your workplace requires it to go back to work in person |  |  |  |  |  |
| If it is available for free at a retail pharmacy (e.g., CVS, Walgreens, etc.) |  |  |  |  |  |
| If it is available for free and delivered by a community health worker to your home/residence |  |  |  |  |  |
| If you have to pay $20 out of pocket to receive it |  |  |  |  |  |
| If your insurance or the government pays you $20 to get the vaccine |  |  |  |  |  |

### Media Sources/Consumption

Q47. How much media coverage of Coronavirus have you been consuming in the past week?

1. Frequent (multiple times a day)
2. A lot (once a day)
3. Somewhat (a few times a week)
4. Very Little (one time or less)

Q49. Of the following TV news media outlets, which would you say is your primary source of news information?

1. Fox News
2. MSNBC
3. CNN
4. Evening news or morning shows on major networks (ABC/NBC/CBS, etc)
5. Alternative news media outlets (e.g., You Tube Channels)
6. Other, specify________
7. No answer

### Personal Health

These questions are borrowed from the Stanford Survey: <https://stanforduniversity.qualtrics.com/jfe/form/SV_0P4AN78jTK29lSB>

Q53. Putting aside Coronavirus, how is your physical health in general?

1. Very good
2. Good
3. Fair
4. Bad
5. Very bad

Q54. Has a doctor ever told you that you have any of the following health conditions [check all that apply]?

- Diabetes
- Heart disease
- Hypertension
- Cancer
- HIV/AIDS
- Asthma
- Chronic lung Disease
- Chronic Obstructive Pulmonary Disease [COPD]
- Overweight/obesity
- Opioid addiction
- Other_______________

Q55. Which of the following best describes your smoking status?

- 1. Current Smoker, frequent
  2. Current Smoker, occasional
  3. Former smoker
  4. Never Smoked

Q57. Have you been engaging in any of the following behaviors more, less or the same as usual since the beginning of the COVID-19 lock-downs in March of this past year?

|  | More the Usual | Same as Usual | Less than Usual | Never do this |
| --- | --- | --- | --- | --- |
| Used pain medications |  |  |  |  |
| Used alcohol |  |  |  |  |
| Smoked cigarettes or vaping products |  |  |  |  |
| Smoked marijuana |  |  |  |  |
| Consumed other drugs/substances |  |  |  |  |
| Got angry and lost my temper with family members or close friends |  |  |  |  |
| Got at least 8 hours of sleep |  |  |  |  |
| Got at least 30 minutes of physical activity a day |  |  |  |  |
| Ate 5 or more fruits and vegetables a day |  |  |  |  |

[modified from [NYC Community Health Survey](https://www1.nyc.gov/assets/doh/downloads/pdf/episrv/chs2018survey.pdf)] Do you have a usual source of medical care (e.g., a clinic, personal doctor or health care provider that you typically go to)?

1. Yes
2. No
3. Unsure

[modified from [NYC Community Health Survey](https://www1.nyc.gov/assets/doh/downloads/pdf/episrv/chs2018survey.pdf)]Was there a time in the past 6 months when you needed medical care but did NOT get it due to concerns about Coronavirus?

1. Yes
2. No
3. Unsure

[modified from [NYC Community Health Survey](https://www1.nyc.gov/assets/doh/downloads/pdf/episrv/chs2018survey.pdf)] Was there a time in the past 6 months when you needed medical care but did NOT get it because you could not afford the out-of-pocket costs, such as copays or deductibles?

1. Yes
2. No
3. Unsure

### Coronavirus Testing/Exposure

In the past 4 weeks have you been…

|  | Yes | No |
| --- | --- | --- |
| …in close contact with a person who has tested positive for Coronavirus? |  |  |
| …ill with a cold or flu-like illness? |  |  |
| …tested for Coronavirus |  |  |

Q61. Have you ever tested positive for Coronavirus?

1. Yes, through a diagnostic test
2. Yes, through an antibody test
3. No

Q61_1. [IF THEY ANSWER A, THAT THEY TESTED POSITIVE] How serious were your symptoms?

1. Very serious
2. Somewhat serious
3. Mild/not serious at all

Q61_2 [IF THEY ANSWER A, THAT THEY TESTED POSITIVE] Please describe anything you would like to here about your symptoms and experience with Coronavirus: ________________________________________________________________________

Q62. Has someone in your immediate family tested positive for Coronavirus since March (either through an antibody test or a diagnostic test)?

1. Yes
2. No

Q63. Has someone in your immediate family died from Coronavirus since the beginning of the pandemic?

1. Yes
2. No

Q64. Has a close friend, colleague or someone in your immediate social circle tested positive for Coronavirus to your knowledge?

1. Yes
2. No

Q65. Has a close friend, colleague or someone in your immediate social circle died from Coronavirus since the beginning of the pandemic?

1. Yes
2. No

### Ideology/Party ID Questions

Q76. In politics today, do you consider yourself a Republican, Democrat, or independent?

1. Democrat
2. Republican
3. Independent
4. Other, describe______________

Q77. If you had to describe your ideology, which of the following would come closest to how you would describe yourself?

1. Liberal
2. Moderate
3. Conservative

Q78. Did you vote in the Democratic Primary?

1. Yes
2. No
3. Not eligible to vote in the Democratic primary
4. Don’t remember

Q79. If you voted in the Democratic Primary, who did you vote for?

1. Bernie Sanders
2. Joe Biden
3. Other, specify_____________
4. Did not vote in the Democratic Primary

Q80. Who did you vote for in the 2016 Presidential Election?

1. Did not vote in the 2016 Presidential election
2. Was not eligible to vote in the 2016 Presidential election
3. Donald Trump
4. Hillary Clinton
5. Someone else, specify_________
6. Don’t remember

Q81. Who did you vote for in the 2020 Presidential election?

1. Joe Biden
2. Donald Trump
3. Write in other candidate, specify___________
4. Did not vote
5. Not eligible to vote

Q82. How did to vote in the 2020 election?

1. In person, early voting
2. In person on election day
3. Mail in ballot
4. Did not vote
5. Not eligible to vote

### Cultural Cognition – 6 Item short form

Q83. Individualism/Communitarianism. People in our society often disagree about how far to let individuals go in making decisions for themselves. How strongly do you agree or disagree with the following statements:

|  | Agree Strongly | Agree | Neither agree nor disagree | Disagree | Disagree Strongly |
| --- | --- | --- | --- | --- | --- |
| ***83***a. The government interferes far too much in our everyday lives |  |  |  |  |  |
| ***83***b. Sometimes government needs to make laws that protect people from hurting themselves |  |  |  |  |  |
| ***83***c. It’s not the government’s business to try to protect people from hurting themselves. |  |  |  |  |  |
| ***83***d. The government should stop telling people how to live their lives. |  |  |  |  |  |
| ***83***e. The government should do more to advance society’s goals even if that means limiting the freedom of choices of individuals. |  |  |  |  |  |
| ***83***f. Government should put limits on the choices individuals can make so they do not get in the way of what is good for society. |  |  |  |  |  |
| ***83g***. People should not do things that are disgusting, even if no one is harmed. |  |  |  |  |  |
| I do whatever I have to in order to work hard and don’t accept hand outs* |  |  |  |  |  |
| In general, I avoid discussions dealing with my feelings and emotions.* |  |  |  |  |  |
| I don't often tell others about my feelings of love and affection for them.* |  |  |  |  |  |
| I don't take orders (or advice) from anybody.* |  |  |  |  |  |

*Masculinity Scale. * Modified from the The Masculine Behavior Scale (MBS) Snell, W. E., Jr. & Snell, W. E., Jr. . (2013) . The Masculine Behavior Scale (MBS) . Measurement Instrument Database for the Social Science. Retrieved from www.midss.ie

Q84. Hierarchy/Egalitarianism. People in our society often disagree about issues of equality and discrimination. How strongly do you agree or disagree with each of these statements.

|  | Agree Strongly | Agree | Neither agree nor disagree | Disagree | Disagree Strongly |
| --- | --- | --- | --- | --- | --- |
| ***84***a. We have gone too far in pushing for equal rights in this country. |  |  |  |  |  |
| ***84***b. Our society would be better off if the distribution of wealth was more equal. |  |  |  |  |  |
| ***84***c. We need to dramatically reduce inequalities between the rich and the poor. |  |  |  |  |  |
| ***84***e. We need to dramatically reduce inequalities between men and women. |  |  |  |  |  |
| ***84***f. Discrimination against minorities is still a very serious problem in our society. |  |  |  |  |  |
| ***84***g. It seems like blacks, women, homosexuals and other groups don’t want equal rights, they want special rights just for them. |  |  |  |  |  |
| ***85h.*** I am proud of my country’s history. |  |  |  |  |  |
| ***86i.*** Chastity is an important and valuable virtue. |  |  |  |  |  |
| ***86j.*** If I were a soldier and disagreed with my commanding officer’s orders, I would obey anyway because that is my duty. |  |  |  |  |  |
| ***86k.*** People should be loyal to their friends or family members, even when they have done something wrong. |  |  |  |  |  |

### Demographics

Q85. What kind of work do you do? That is, what was your occupation?
 (For example: plumber, typist, farmer. Housewife, student..) ___________________________________

Q86. Is your job considered “essential” according to the rules of your state or what your employer has told you?

1. Yes
2. No

Q87. What year were you born? ________

Q88. Please enter your zip or postal code of your permanent address: __________

[From BRFSS] Do you rent or own your current home?

- - 1. Rent
    2. Own
    3. Some other arrangement (such as living in a group home or staying with friends/family without paying rent)

Q89. Were you born in the US?

1. Yes
2. No

(Question wording from Pew: <https://www.pewforum.org/wp-content/uploads/sites/7/2015/11/201.11.03_rls_ii_questionnaire.pdf>)

Q91. What is your present religion, if any?

1. Protestant (Baptist, Methodist, Non-denominational, Lutheran, Presbyterian, Pentecostal, Episcopalian, Reformed, Church of Christ, etc.)
2. Roman Catholic (Catholic)
3. Mormon (Church of Jesus Christ of Latter-day Saints/LDS)
4. Orthodox (Greek, Russian, or some other orthodox church)
5. Jewish (Judaism)
6. Muslim (Islam)
7. Buddhist
8. Hindu
9. Atheist (do not believe in God)
10. Agnostic (not sure if there is a God)
11. Something else (SPECIFY)_________

Q92. How important is religion in your life – very important, somewhat important, not too important, or not at all important?

1. Very important
2. Somewhat important
3. Not too important
4. Not at all important
5. Don’t know/Refused

Q93. What gender do you identify as?

1. Female
2. Male
3. Other, ___________

Q94. What is the highest level of school you have completed?

1. Less than high school
2. High school
3. College or University
4. Graduate degree

How often do you take public transportation?

1. Daily
2. Weekly
3. One in awhile
4. Never

Q95. What was your annual household income in 2019 in US dollars?

1. < $10,000
2. $10,001-$20,000
3. $20,001-$50,000
4. $50,001-$75,000
5. $75,001-$150,000
6. $150,001-$200,000
7. $200,000-$250,000
8. >$250,000

Q96. How would you describe your class background as you experienced it, growing up?

1. Lower class/poor
2. Lower middle class
3. Middle class
4. Upper-middle class
5. Upper-class/wealthy

Q97. Are you Hispanic or Latino?

- 1. Yes
  2. No

Q97a. What national origin do you identify with?

1. Puerto Rican
2. Mexican
3. Dominican
4. Cuban
5. Other, specify in the box below
6. None

Q98. Which one or more of the following would you say best describes your race? [check all that apply]^[[1]](#endnote-1)^

- White
- Black or African American
- Asian
- American Indian or Alaskan Native
- Middle Eastern or North African
- Native Hawaiian or Other Pacific Islander
- Some other Race Ethnicity or Origin

Thank you for answering these questions. Before you complete the survey, please take a moment to read the following debriefing message.

Debrief: In order to properly test our hypotheses about how different types of information about who will get priority for a COVID-19 vaccine affects willingness to vaccinate, mask wearing, and social distancing of New Yorkers during the pandemic, we created a series of fictitious newspaper articles and hypothetical scenarios. The article at the beginning of the survey that you may have read and the two sets of scenarios about individual vaccination preferences and vaccine requirement policies were designed for this purpose. The information presented in the article was correct, but we altered the way it was presented in order to test hypotheses about the credibility of these types of messages. The scenarios in the questions asking about the willingness to take a COVID-19 vaccine and support for the vaccine requirement policy were hypothetical. We chose not to tell you this at the time in order to ensure that your reactions to the information presented in the article were spontaneous and were not influenced by your prior knowledge about the purpose of this study.

You can view the original press release on which this article was based by clicking here:

<https://www.latimes.com/science/story/2020-10-03/who-will-get-priority-for-covid-19-vaccines>

The information we collected from this survey will be maintained anonymously. Names and other identifying information will not be used in any presentation or paper written about this project.

If you have any questions or concerns about this study please contact: XXX.

1. New census categories: <https://www.pewresearch.org/fact-tank/2015/06/18/census-considers-new-approach-to-asking-about-race-by-not-using-the-term-at-all/> [↑](#endnote-ref-1)
