# Supplementary material for: Does highlighting COVID-19 disparities reduce or increase vaccine intentions? evidence from a survey experiment in a diverse sample in New York State prior to vaccine roll-out
Source: PLoS One. 2022 Dec 14;17(12):e0277043. doi: 10.1371/journal.pone.0277043 (PMC9750017; doi:10.1371/journal.pone.0277043)
Supplement: S3 File — (DOCX) [file pone.0277043.s003.docx]

# Code for supporting materials is available at: https://github.com/TheYongjinChoi/NYS-COVID19-Disparities-Survey
