# Supplementary material for: Does highlighting COVID-19 disparities reduce or increase vaccine intentions? evidence from a survey experiment in a diverse sample in New York State prior to vaccine roll-out
Source: PLoS One. 2022 Dec 14;17(12):e0277043. doi: 10.1371/journal.pone.0277043 (PMC9750017; doi:10.1371/journal.pone.0277043)
Supplement: Attachment — Submitted filename: Response to Reviewers.docx [file pone.0277043.s004.docx]

May 2, 2022

Dear Reviewers,

Thank you for providing us with these helpful comments on our paper, which have allowed us to substantially strengthen and update the manuscript. Some of the issues that you raised about the literature review being out of date and the overall contribution have, at least in part, to do with the lengthy peer-review process that the article has gone through, which have made the findings less timely than when we first undertook the research. Nonetheless, we do believe that the research community is better off having access to our research and its approach than not- for instance to be able to replicate the results or try similar experiments in different samples. Throughout we have tried to update the manuscript, better clarify what we see as the main contributions of the project and be more forthright about some of its limitations. We have substantially revised the manuscript throughout.

Below we outline our response to your comments in red font.

Sincerely,

The Authors

Reviewers' comments:

5. Review Comments to the Author

Please use the space provided to explain your answers to the questions above. You may also include additional comments for the author, including concerns about dual publication, research ethics, or publication ethics. (Please upload your review as an attachment if it exceeds 20,000 characters)

Reviewer #1: This is an interesting study that examines vaccine hesitancy using a mixed methods approach combining survey analysis, qualitative data analysis of open-ended questions, and a survey experiment. In spite of the strengths of the methods and analysis, there are a number of problems with the manuscript that lead me not to recommend it for publication in its present form.

(1) The literature review does not sufficiently examine results of other published studies examining COVID-19 vaccine hesitancy in the US over the past year. Situating the present findings in light of more data from around the US as well as comparisons to different time periods of the pandemic would be very important to get a better sense of how to interpret the findings. E.g., the present study seems to support other findings about Republicans and African Americans expressing more hesitancy. But have other studies similarly found women to be more hesitant than men or older people more likely to be hesitant, or are the findings elsewhere different? See for a review of some of this literature: Wang, Ying, and Yu Liu. "Multilevel determinants of COVID-19 vaccination hesitancy in the United States: a rapid systematic review." Preventive Medicine Reports (2021): 101673.

Thank you for noting this. We agree that our literature review section needs to be updated to reflect current knowledge of the COVID-19 vaccine landscape in the US. We have tried to update and synthesize throughout the introduction including incorporating the helpful review article you cite (Wang, Ying, and Liu, 2021). We have substantially updated the literature throughout and tried to better situate the findings in the time period in which the survey/survey experiment was undertaken (Nov/Dec 2020 prior to vaccine roll-out and just following the 2020 Presidential elections).

(2) The study suffers from serious limitations regarding generalizability, due to the time period of the data collection as well as sampling methodology. Neither the abstract nor the title notes that the study is focused on a non-representative sample of New York State residents during November-December 2020, which is before the COVID-19 vaccine was approved. There are serious limitations with what we can learn about vaccine hesitancy from this study.

We do not dispute this point about the limitations of what we can learn from this study, including that it is from a non-representative sample of New York State residents and at a single time-point in the pandemic before vaccines were available to everyone. However, we would like to clarify that the study was undertaken primarily as a survey experiment and to collect short qualitative information on reasons that people give for desiring/hesitating to vaccinate, neither of which require representative samples to make broader inferences.

We have tried make these points about its contribution more salient throughout, including acknowledging this in the limitations section.

We have also revised the title to better signal what we view as the main contribution of this piece (i.e., the survey experiment and qualitative findings) and the specificity of the sample/time period. We have revised the title as follows:

“Does Framing COVID-19 in Terms of Disparities Reduce or Increase Vaccine Hesitancy? Evidence from a Survey Experiment in a Diverse Sample in New York State prior to vaccine roll-out”

Finally, we have reorganized our presentation of the order of our results, starting with the survey experiment followed by the descriptive survey analysis and the qualitative results. We hope these revisions can better contextualize the contribution with greater emphasis on the survey experiment.

First, even though NYS is demographically and politically diverse, there’s no weighting applied in this dataset that could allow the sample to be generalized to the state population. Oversampling blacks and Hispanics is great, but again without sample weights, the results of a similar analysis may look very different in a weighted sample. The paper needs to also say something about how the results of a similar study conducted elsewhere in the US might look.

Second, the time period is an important issue. After the approval of the vaccine, vaccination attitudes changed considerably over the span of a few months as more information (and misinformation) about mRNA vaccines started to circulate. mRNA vaccines have introduced new kinds of fears and hesitancies which are crucial for us to understand for the very reasons articulated in the manuscript, but the study is unable to offer such insights. It is also the case that since early 2021, African American communities have taken leadership in driving vaccination efforts, even using churches as vaccination centers. So would the same study, if carried out four or six months later, have yielded different results? This is an important consideration that the authors need to address.

This is not to say the study cannot make important contributions; they are just narrower in scope and need to be stated as such.

We have tried to tone done the contributions in various places, starting with the title, which we renamed “Does Highlighting COVID-19 Disparities Reduce or Increase Vaccine Intentions? Evidence from a Survey Experiment in a Diverse Sample in New York State Prior to Vaccine Roll-out.” We now discuss more about evolving trends in hesitancy and vaccine uptake based on updated literature review. Further, by emphasizing the survey experiment, we are contributing more broadly to emerging literature on whether messages emphasizing disparities are harmful or helpful to vaccine promotion. We believe that even though our experimental results on this front are null, it is important to report this.

Minor points:

The coding of the qualitative data needs to distinguish between distrust or refusal of vaccines in general vs. distrust of this specific vaccine (e.g., “I don’t believe in getting that vaccine”, “I do not trust the vaccine”). Those seem to be conflated in the current study. It is also difficult to tell how certain statements suggesting conspiracy theories (e.g., concerns about microchips) do not also underlie some of the vaguer statements of distrust (i.e., there may be more conspiracy theorists who just didn’t articulate their reasons). Also given the non-representativeness of the sample, I’m not sure what to make of the distributions in the qualitative data; just because a particular theme stands out in this dataset, that doesn’t mean the opinion it reflects is proportionately prevalent in the broader population.

Thank you for this comment. We have made several adjustments to the paper to make our qualitative work clearer, including writing out the precise questions we posed in the methods and rewriting our section on the Qualitative results- see p. 24-26. We also took out the Figures and put those in the Supplementary materials for those who want more details on relative frequencies of statements.

Regarding the experiment, I found it striking (and this is not mentioned in the manuscript at all) that in the image of the African American person receiving the vaccine, the person administering the vaccine is white. If part of the reason for vaccine hesitancy is ongoing racism experienced by blacks in the healthcare system, then this prime would only reinforce such mistrust. Might the results have been different if the physician in the image had been black? Did the researchers conduct any cognitive testing of these images in order to make sure they did not have unintended effects/interpretations?

Yes, this was an oversight on our part. The image was the actual image associated with the newspaper article that we built the experiment from, but we could have been more thoughtful about whether the racial discordance in the image would work against our hypotheses. We agree that the experiment would have benefited from cognitive testing of these images, but we did not undertake this. We have now listed this as a limitation in the Limitations section and something that future research should consider if they try to replicate this experiment or related experiments.

See Limitations section, p. 31: “Future studies that may wish to replicate this experiment with a racially concordant doctor and patient or related experiments could consider similar designs using racially concordant doctors and patients to mitigate this possible source of bias. Pre-testing of the image could have helped to assess how the racial discordance plays into the overall message.”

Reviewer #2: Thank you for the opportunity to review this article. 

I believe the topic is important and that differential uptake in COVID-19 vaccination will exacerbate pre-existing socioeconomic inequalities amongst marginalised ethnic and social groups. This study adds to our understanding or confirms our knowledge to an extent. Many thousands of unvaccinated people have died in the USA and elsewhere despite the availability of safe, effective vaccines that can significantly reduce the risk of serious disease and death. The topic is therefore important, timely and helpful. 

General feedback: the article lacks clarity in places. The authors also assume that the readers will not exactly what they are talking about but of course, this should not be assumed and the language should be made accessible. The authors just mention terms like 'vaccine hesitancy' without even an attempt to explain what it means. It should be defined and an appropriate citation is given. Acronyms are widely used without expanding what they stand for in the main body of the article. For example, in the introduction, it says NH Black. I understand that the authors have explained in the abstract but this should be done in the introduction as well. Non-Hispanic is one word but it will add to the clarity. I don't think ASAP is an appropriate acronym in an academic paper and should not be used. 

Thanks for these points. It is true that we perhaps assumed a level of knowledge of vaccine hesitancy that may not be appropriate for a more general readership journal like PLoS One. We have added a definition of vaccine hesitancy to the introductory paragraph. We have also increased our own precision by clarifying that we are actually measuring intent to vaccinate against COVID-19. We have also spelled out the abbreviations the reviewer mentions (non-Hispanic and as soon as possible). In general, we have substantially re-written the article to the point where the track changes shown that a large portion of the manuscript has been completely rewritten and updated.

I have the following specific feedback for the authors. 

Abstract:It is not clear what the authors mean by 'the experimental manipulation of a vaccine promotion message'. This needs to be written more clearly in the abstract without having to look at the methodology and results for what they mean. Where the authors mention 'ascriptive' they need to open a bracket and list some of those characteristics. For example ascriptive characteristic (i.e., race, gender....).

Thanks for these suggestions. We have amended the sentence in the abstract as follows:

“Experimental manipulation of a racial equity vaccine promotion message via an online survey experiment had no effect on intention-to-vaccinate in the full sample or in racial, ethnic and partisan subsamples.”

We have also clarified the term “ascriptive characteristics” per the Reviewer’s suggestion in the Highlights and in other places where it is mentioned in the manuscript. Ascriptive characteristics refer to people’s “outward identities” as opposed to underlying values traits/personality characteristics.

Ethics:I am not sure why the institutional review board is anonymised. For a survey of this nature, I believe we more information about ethics and the approval reference. 

This was originally done for anonymous peer review purposes, but we have now added more detailed institutional review board information in the text now:

p. 9: “This study was approved by the Institutional Review Board of the University at Albany (IRB study number, 20X258).”

Methods:
The regression analyses in controlling for potential confounding factors and adjusting for demographic characteristics are good and improve the validity and reliability of the results. 

Results:
The odds ratio in NH blacks and to a certain extent Hispanics and NH Whites Trump voters are quite high and significant. The statistical analyses look fine to me but will need to be double-checked by a statistician. 

There is a very high level of vaccine hesitancy in women in this sample. Were these reproductive-aged women? Low intent to vaccinate is particularly high in pregnant or reproductive-aged women as multiple studies have shown. It would be important to comment on that given how serious COVID-19 infection is in pregnancy.

Heightened vaccine hesitancy in women has been established in other studies, including the review article by Wang & Li that we now cite. Sex/gender was not our main focus here but we have updated the literature we cite in regards to this finding throughout.

Free text responses - the authors rightly acknowledge that they could not do proper qualitative analyses on these. I believe it is not right to impose a quantitative framework on a qualitative response. It does not matter what percentage of people mentioned lack of trust it is the depth, richness, and complexity are would be uncovered through qualitative responses.

Thank you for this comment. We believe our analysis is appropriate to the type of data we have. In terms of presentation, we think there is value in providing the reader a sense between a much-mentioned and a once-mentioned sentiment. Nevertheless, we have greatly revised this section to emphasize the common themes that emerged and put the Figures in the Supplemental materials for those who want to know more about how we arrived at our conclusions. We have greatly revised out qualitative results section. See p. 24-26.

Discussion:

The limitations section is adequate. 

The authors say that higher vaccine hesitancy among Non-Hispanic Blacks is not entirely accounted for institutional mistrust compared to NH Whites Trump votes. However, it would be important to discuss from existing literature other possible reasons for this.

We have elaborated this point further in the Discussion on p. 19 as follows:

“Unlike vote choice, the significance of race and ethnicity persisted even after the inclusion of measures of mistrust, alternative media use, and individualism. It is possible that the particular measures of mistrust were insufficient to capture the nuanced mechanisms through which distrust in these institutions’ shapes COVID-19 vaccine attitudes. Prior studies have found that mistrust among Black populations in the U.S. may be further exacerbated by the general sociopolitical climate in the US [53], or potential confounding with socioeconomic status that is difficult to measure accurately and adjust for in observational studies with online samples (e.g., lack of financial resources or housing insecurity) [54].”

Reviewer's Responses to Questions

**Comments to the Author**

1. Is the manuscript technically sound, and do the data support the conclusions?

The manuscript must describe a technically sound piece of scientific research with data that supports the conclusions. Experiments must have been conducted rigorously, with appropriate controls, replication, and sample sizes. The conclusions must be drawn appropriately based on the data presented.

Reviewer #1: Partly

Reviewer #2: Yes

2. Has the statistical analysis been performed appropriately and rigorously?

Reviewer #1: Yes

Reviewer #2: Yes

3. Have the authors made all data underlying the findings in their manuscript fully available?

The  [PLOS Data policy](http://www.plosone.org/static/policies.action#sharing) requires authors to make all data underlying the findings described in their manuscript fully available without restriction, with rare exception (please refer to the Data Availability Statement in the manuscript PDF file). The data should be provided as part of the manuscript or its supporting information, or deposited to a public repository. For example, in addition to summary statistics, the data points behind means, medians and variance measures should be available. If there are restrictions on publicly sharing data—e.g. participant privacy or use of data from a third party—those must be specified.

Reviewer #1: Yes

Reviewer #2: Yes

4. Is the manuscript presented in an intelligible fashion and written in standard English?

PLOS ONE does not copyedit accepted manuscripts, so the language in submitted articles must be clear, correct, and unambiguous. Any typographical or grammatical errors should be corrected at revision, so please note any specific errors here.

Reviewer #1: Yes

Reviewer #2: No

6. PLOS authors have the option to publish the peer review history of their article ([what does this mean?](https://journals.plos.org/plosone/s/editorial-and-peer-review-process#loc-peer-review-history)). If published, this will include your full peer review and any attached files.


If you choose “no”, your identity will remain anonymous but your review may still be made public.


**Do you want your identity to be public for this peer review?** For information about this choice, including consent withdrawal, please see our [Privacy Policy](https://www.plos.org/privacy-policy).

Reviewer #1: No

Reviewer #2: No

[NOTE: If reviewer comments were submitted as an attachment file, they will be attached to this email and accessible via the submission site. Please log into your account, locate the manuscript record, and check for the action link "View Attachments". If this link does not appear, there are no attachment files.]

While revising your submission, please upload your figure files to the Preflight Analysis and Conversion Engine (PACE) digital diagnostic tool, <https://pacev2.apexcovantage.com/>. PACE helps ensure that figures meet PLOS requirements. To use PACE, you must first register as a user. Registration is free. Then, login and navigate to the UPLOAD tab, where you will find detailed instructions on how to use the tool. If you encounter any issues or have any questions when using PACE, please email PLOS at [figures@plos.org](mailto:figures@plos.org). Please note that Supporting Information files do not need this step.
